# Low concentration of heparin used for permanent catheters canal locking is effective and diminishes the risk of bleeding

**DOI:** 10.1007/s11255-012-0151-y

**Published:** 2012-03-15

**Authors:** Tomasz Hryszko, Szymon Brzosko, Michal Mysliwiec

**Affiliations:** Department of Nephrology and Transplantation, Medical University of Bialystok, Ul. Zurawia 14, 15-540 Bialystok, Poland

**Keywords:** Bleeding, Hemodialysis, Heparin, Permanent catheter

## Abstract

**Purpose:**

There is an increasing number of patients being dialyzed with permanent catheters (PC). In the majority of cases, heparin is used to maintain PC patency. This practice causes clotting disturbances due to heparin leakage and may predispose the patient to bleeding episodes. It has not been well studied whether lowering heparin concentration for canal locking decreases short-term bleeding complications after PC placement.

**Methods:**

This was a prospective single-center randomized open-label trial conducted in hemodialyzed patients undergoing PC insertion. Low concentration of heparin (LCH) 2,500 IU/ml versus high concentration of heparin (HCH) 5,000 IU/ml was randomly used for catheter lumens locking. The primary endpoint was the occurrence of bleeding within 24 h after catheter placement. The effects of clinical and laboratory data on bleeding events were analyzed as secondary endpoints.

**Results:**

Seventy-five patients (37 in LCH) were enrolled in the study. Only in the HCH group we found a significant prolongation of activated partial thromboplastin time (APTT) 2 h after PC placement (*p* < 0.001). There was a higher number of bleeding episodes in the HCH group (n = 16; 42.1%) than in the LCH group (n = 7; 18.9%) (χ^2^ = 4.74; *p* = 0.029). In univariate analysis, assignment to HCH, baseline APTT, use of low molecular weight heparin, and femoral localization were associated with bleeding events. In multivariate analysis, the use of HCH (odds ratio [OR] 3.64; 95% confidence interval [95% CI] 1.10–12.05) and baseline APTT (OR 1.12; 95% CI 1.002–1.250) predicted bleeding after PC insertion.

**Conclusion:**

LCH used for canals locking decreases bleeding events in the first 24 hours after permanent catheter placement, compared to HCH.

## Introduction

The mainstay of successful hemodialysis therapy is reliable vascular access. Nowadays, two viable options exist for hemodialysis patients (HD): arteriovenous fistula or central venous catheter. The number of patients dialyzed with a central venous catheter gradually continues to increase [1, 2], even though current guidelines unequivocally recommend an arteriovenous fistula as the preferred vascular access for HD [3]. In dialysis centers, up to 80% of HD start renal replacement therapy with a catheter [4] and in one-third of them it serves as a long-term vascular access [5]. Both patient- and nephrologist-related factors are responsible for this phenomenon [6, 7]. It may be anticipated that this trend will not reverse in the near future, thus nephrologists will be facing patients needing permanent catheter (PC) insertion more and more frequently.

PC placement is a relatively safe procedure, although sometimes it is associated with the development of several complications. The spectrum of possible complications varies from life-threatening like cardiac tamponade or pneumothorax, to minor such as a puncture of an artery with a “finder needle.” One of the possible problems often encountered after PC placement is bleeding from the site of insertion. It is mainly caused by clotting derangement due to the leak of heparin from the catheter lumens [8]. The concentration of heparin instilled into PC canals varies from 1,000 to 10,000 IU/ml, as there are no guidelines addressing this problem.

It was shown earlier [9] that heparin concentration did not adversely affect catheter patency. The aim of the study was to test a hypothesis that the lower heparin concentration used for canal locking limits bleeding episodes in HD after PC insertion.

## Subjects and methods

This was a single-center, prospective, randomized, open-label trial evaluating the influence of heparin concentration used for permanent catheters canals locking on frequency of bleeding complications in HD. Patients were randomized with toss a coin method into one of two groups, which differed in concentration of heparin (2,500 IU/ml low concentration of heparin—LCH vs. 5,000 IU/ml high concentration of heparin—HCH) used for catheter lumens locking.

All patients received dual lumen cuffed catheters (Covidien Palindrome, Mansfield, MA, USA). The catheter insertion took place in a separate room, under aseptic conditions and local anesthesia (1% lidocaine). The site of insertion was based on clinical factors and was left to the operator’s decision. Catheters 14.5 Fr/28 cm in length were placed in the internal jugular vein or 14.5 Fr/55 cm when femoral vein was used. Immediately after catheter placement, its patency was checked and lumens were filled with nominal volume of heparin at a concentration in accordance with the assigned group (2,500 or 5,000 IU/ml). The correct localization of the catheter was confirmed with X-ray.

Before catheter placement, white blood cells (WBC) and platelet count (PLT), hemoglobin (HGB), fibrinogen (FBG) concentration, international normalized ratio (INR), and activated partial thromboplastin time (APTT) were measured with standard laboratory methods and demographic data were recorded. Two hours after the procedure, APTT was determined. All patients were observed for 24 h.

Before entering the study, all patients gave informed consent. The study was conducted in accordance with the principles of the Declaration of Helsinki. The research protocol was reviewed and accepted by a local ethics committee.

### Definitions

The indication for permanent catheter insertion was classified as: incident renal failure—the catheter was placed, when there was a need for dialysis initiation and the patient did not have a-v fistula and prevalent renal failure—the catheter was inserted in chronically dialyzed patient with dysfunctional fistula.

The causes of end stage renal disease (ESRD) were divided into: glomerulonephritis, diabetic nephropathy, autosomal polycystic kidney disease, and others (unknown, obstructive nephropathy).

Bleeding episode was defined as the need to change dressings once or more within 24 h after catheter insertion. After catheter insertion, the site of surgery was observed for 60 s to assure that there is no persistent bleeding from the wound.

### Statistics

Data are presented as mean ± SD and frequency percentage for categorical variables.

In 4 cases APTT 2 h after insertion was immeasurable—blood did not clot. These cases were treated as APTT equal to 163.7 s—the longest time, which was measured in the cohort during the study.

Independent or dependent continuous variables were compared with an appropriate variant of Student *t* test. Differences between categorical data were evaluated with χ^2^ test. For bivariate associations between bleeding episodes and variables of interest, logistic regression analysis was used. The variables associated with endpoint in univariate analysis (*p* < 0.10) were included into multivariate logistic regression analysis.

A two-tailed *p* value below 0.05 was considered significant. All computations were performed with Statistica 9.1 for Windows (StatSoft Inc.; Tulsa, OK, USA).

## Results

Seventy-five patients (36 males, 48%) were enrolled in the study. The mean age of studied population was 64.5 ± 14.5 years. The following were causes of ESRD in the studied population: glomerulonephritis (*n* = 20; 27%), diabetic nephropathy (*n* = 12; 16%), autosomal polycystic kidney disease (*n* = 10; 13%), and others (*n* = 33; 44%).

Thirty-eight patients (50.7%) were assigned to the HCH group. Internal jugular vein was used in 33 (89%) patients in LCH and 31 (82%) in HCH group (*p* = 0.35). The catheter was placed on the right side in 27 (73%) subjects in LCH and 34 (90%) in HCH (*p* = 0.06). Demographic and laboratory data of both groups are presented in Table 1. At baseline, both groups did not differ significantly in any of evaluated parameters.

**Table 1:** Characteristic of two analyzed groups

	LCH	HCH
Subjects (*n*)	37	38
Female/male	18/19	21/17
DM (*n*; %)	9; 24	8; 21
Cause of catheter insertion: incident/prevalent renal failure	10/27	12/26
Age (years)	64 ± 15	65 ± 14
ASA (*n*; %)	10; 28	9; 26
LMWH (*n*; %)	3; 8	3; 8
HGB (g/dl)	10.2 ± 1.9	10.3 ± 1.8
PLT (×10^3^/µl)	215.1 ± 67.5	215.3 ± 99.4
FBG (mg/dl)	431.7 ± 200.7	424.7 ± 203.4
APTT (s)	38.8 ± 5.9	38.9 ± 5.3
INR	1.09 ± 0.11	1.08 ± 0.11

*APTT* activated partial thromboplastin time, *ASA* use of acetylsalicylic acid, *FBG* fibrinogen, *HCH* high concentration of heparin, *HGB* hemoglobin, *INR* international normalized ratio, *LCH* low concentration of heparin, *LMWH* use of low molecular weight heparin, *PLT* platelet count

**Fig. 1:**
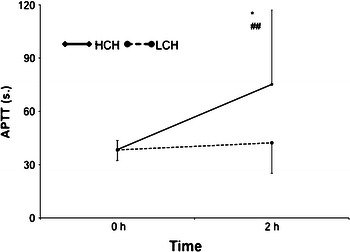
APTT values with regard to heparin concentration used for catheter canal locking. (*Baseline APTT vs. APTT 2 h after catheter insertion *p* < 0.001, ^##^comparison of APTT 2 h after catheter insertion HCH vs. LCH *p* < 0.001). *APTT* activated partial thromboplastin time, *HCH* high concentration of heparin, *LCH* low concentration of heparin

Two hours after the catheter placement, there was significant prolongation of APTT value only in HCH group (from 38.5 ± 5.1 to 75.2 ± 41.8 s; *p* < 0.001). In LCH, there was slight non-significant prolongation of APTT (from 38.3 ± 6.0 to 42.3 ± 17.2 s; *p* = 0.17). There was striking difference in APTT value between LCH and HCH group 2 h after catheter insertion (42.3 ± 17.2 vs. 75.2 ± 41.8 s; *p* < 0.001) (Fig. 1).

**Fig. 2:**
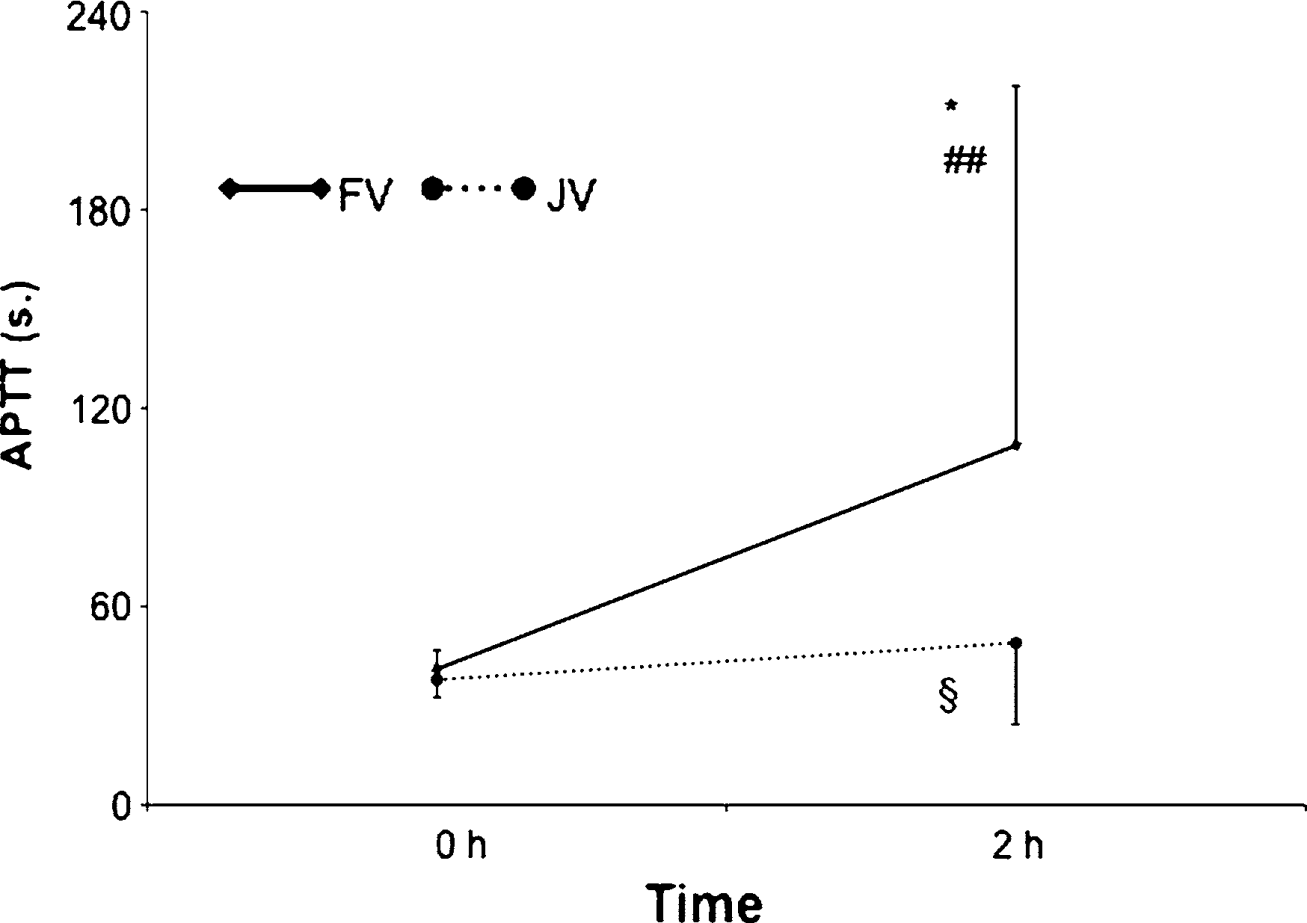
APTT values with regard to the length of catheter used (*Baseline APTT vs. APTT 2 h after catheter insertion in FV *p* < 0.01, ^§^baseline APTT vs. APTT 2 h after catheter insertion in JV *p* < 0.01, ^##^comparison of APTT 2 h after catheter insertion FV vs. JV *p* < 0.001) *APTT* activated partial thromboplastin time, *JV* jugular vein, *FV* femoral vein

Insertion of catheter into jugular or femoral vein caused significant prolongation of APTT irrespectively of heparin concentration used for canal locking (Jugular Vein; from 37.86 ± 5.3 to 48.99 ± 24.7 s; *p* = 0.002, Femoral Vein; from 41.06 ± 5.6 to 108.7 ± 51.2; *p* = 0.001) (Fig. 2).

There was higher number of bleeding episodes in HCH (16; 42.1%) than in LCH (7; 18.9%) (χ^2^ = 4.74; *p* = 0.029). During the study, there were no cases of catheter thrombosis in either LCH or in HCH.

In univariate logistic regression, the use of LMWH (χ^2^ = 3.35; *p* = 0.07), assignment to HCH group (χ^2^ = 4.55; *p* = 0.033), femoral localization (χ^2^ = 3.22; *p* = 0.073), baseline APTT value (χ^2^ = 5.65; *p* = 0.02), and APTT 2 h after catheter insertion (χ^2^ = 9.26; *p* = 0.002) were associated with bleeding episodes (Table 2).

**Table 2:** Results of univariate logistic regression analysis

Univariate analysis			
Variable	OR	95% CI	*p*
Sex	1.27	0.47–3.46	0.63
Age	1.01	0.97–1.05	0.60
Indication for catheter insertion: incident/prevalent renal failure	0.79	0.25–2.53	0.69
ASA use	0.95	0.30–3.04	0.93
LMWH use	5.26	0.86–32.09	0.07
HCH group assignment	3.12	1.08–9.02	0.03
Femoral localization	3.32	0.88–12.57	0.07
Side of catheter insertion	1.33	0.35–4.99	0.67
HGB	1.10	0.83–1.44	0.50
PLT	1.00	0.99–1.01	0.88
FBG	1.00	0.99–1.00	0.15
Baseline APTT	1.13	1.02–1.25	0.02
Baseline INR	0.46	0.01–39.74	0.73
APTT 2 h after catheter insertion	1.03	1.01–1.05	0.002

*APTT* activated partial thromboplastin time, *ASA* use of acetylsalicylic acid, *CI* confidence interval, *FBG* fibrinogen, *HCH* high concentration of heparin, *HGB* hemoglobin, *INR* international normalized ratio, *LMWH* use of low molecular weight heparin, *OR* odds ratio, *PLT* platelet count

As the APTT value 2 h after insertion was the consequence of group assignment, two multivariate models were built in which group assignment (Model 1) or APTT value 2 h after insertion (Model 2) was used as an independent variable. In Model 1 (χ^2^ = 16.85; *p* = 0.002), group assignment and baseline APTT value were the only predictors of bleeding occurrence. Model 2 (χ^2^ = 19.86; *p* = 0.00053) revealed just APTT value 2 h after insertion as the independent predictor of bleeding after catheter insertion (Table 3).

**Table 3:** Results of multivariate analysis

Variable	OR	95% CI	*p*
Model 1 χ^2^ = 16.85; *p* = 0.002			
HCH assignment	3.64	1.10–12.05	0.03
Baseline APTT	1.12	1.002–1.250	0.04
LMWH use	5.94	0.82–42.88	0.07
Femoral localization	3.12	0.73–13.32	0.12
Model 2 χ^2^ = 19.86; *p* = 0.0005			
APTT 2 h after catheter insertion	1.03	1.01–1.06	0.01
Baseline APTT	1.10	0.99–1.23	0.07
LMWH use	4.38	0.61–31.55	0.14
Femoral localization	0.72	0.09–5.62	0.75

Model 1 included use of LMWH, site of catheter placement, baseline APTT value, and group assignment. Model 2 included instead of group assignment value of APTT 2 h after catheter insertion

*APTT* activated partial thromboplastin time, *CI* confidence interval, *HCH* high concentration of heparin, *LMWH* use of low molecular weight heparin, *OR* odds ratio

## Discussion

According to obtained results, lower heparin concentration decreases the odds of bleeding after PC insertion. As current guidelines do not give any recommendations on this subject, results of this study may give a rationale to routinely use lower heparin concentration to fill lumens of PC at least just after insertion. Our results are in line with the retrospective study by Yevzlin et al. [10], who also showed that concentrated heparin lock is associated with major bleeding events after PC placement.

The bleeding tendency in patients with PC results from coagulation abnormalities due to heparin used for catheter lumens locking. In our study APTT 2 h after insertion was prolonged ~2 times, if the high concentration of heparin was used. It was reported earlier that significant APTT prolongation occurs just after 10 min of canals locking with heparin [11, 12]. The observed systemic anticoagulation is caused by at least 3 mechanisms. Firstly, there are reports showing that real volume of catheter lumens is often substantially lower then that reported by the manufacturer [11]. Secondly, heparin diffuses from catheter canals into systemic circulation within an hour after placement, and thirdly, the fast push of the heparin is associated with a higher degree of clotting abnormalities [8].

Placement of PC into the femoral vein was associated with bleeding events in our study. Up to our knowledge, there are no data linking the site of catheter insertion with the risk of bleeding complications. As in our study all catheters inserted into the femoral vein were almost two times longer than those placed in the jugular vein, the heparin volume was also substantially larger. Thus, we may assume that the higher amount of heparin leaked out from the catheter. Indeed, comparison of APTT prolongation between femoral and jugular catheters showed a striking difference in the magnitude of clotting disturbances. This hypothesis was validated by the results of multivariate analysis. In both models, femoral localization was not significantly associated with the primary endpoint. Thus, it seems that the instilled volume is the culprit of observed associations, but not the site of catheter insertion.

Another important point to be considered is the question, does the lower heparin concentration increase the risk of catheter thrombosis? According to the obtained results, the risk of catheter thrombosis was equal between both groups but we have to bear in mind that the observation period in our study was just 24 h. Noteworthy, it may be speculated based on earlier data that heparin at a concentration from 1,000 to 2,500 IU/ml is equally effective as heparin used at a higher concentrations in prevention of catheter thrombosis [9, 12]. Thus, it seems that with lower heparin concentration the same catheter patency is obtained.

The study has potential limitations, such as small number of studied patients, not blinded design, and short observation period. Although it is to be underlined that during the design of the study the primary aim was to evaluate the bleeding rates within the immediate post surgery period as it often prolongs hospitalization associated with PC placement, utilizes additional resources, and finally increases the overall costs. We are aware that a larger trial is needed in order to draw a final conclusion.

In conclusion, locking of permanent catheters lumens with heparin at 2,500 IU/ml when compared to 5,000 IU/ml decreases the risk of bleeding complications in the post surgery period without compromising their patency.
